# 8-Methyl-4-morpholinoethyl-1-thia-4-aza­spiro­[4.5]decan-3-one

**DOI:** 10.1107/S1600536808022459

**Published:** 2008-07-23

**Authors:** Mehmet Akkurt, Şerife Pınar Yalçın, Nalan Terzioğlu Klip, Orhan Büyükgüngör

**Affiliations:** a Department of Physics, Faculty of Arts and Sciences, Erciyes University, 38039 Kayseri, Turkey; b Department of Pharmaceutical Chemistry, Faculty of Pharmacy, İIstanbul University, Beyazıt 34116, Istanbul, Turkey; c Department of Physics, Faculty of Arts and Sciences, Ondokuz Mayıs University, 55139 Samsun, Turkey

## Abstract

In the title compound, C_15_H_26_N_2_O_2_S, the cyclo­hexane and morpholine rings adopt chair conformations, while the thia­zole ring has a twist conformation. An intra­molecular C—H⋯S hydrogen-bond inter­action forms a five-membered ring. The crystal packing involves C—H⋯O=C inter­molecular inter­actions where carbonyl O atoms act as double acceptors to two symmetrically related H atoms.

## Related literature

For general background, see: Andres *et al.* (2000 ▶); Vicini *et al.* (2006 ▶); Küçükgüzel *et al.* (2002 ▶); Barreca *et al.* (2001 ▶); Rao *et al.* (2004 ▶); Gududuru *et al.* (2004 ▶). For related literature, see: Akkurt *et al.* (2007 ▶, 2008 ▶). For bond-length data, see: Allen *et al.* (1987 ▶). For ring conformation puckering parameters, see: Cremer & Pople (1975 ▶).
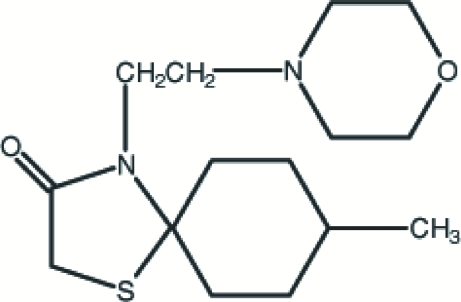


## Experimental

### 

#### Crystal data

C_15_H_26_N_2_O_2_S
*M* *_r_* = 298.45
Triclinic, 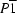

*a* = 7.8629 (4) Å
*b* = 10.5239 (6) Å
*c* = 10.8252 (6) Å
α = 94.974 (5)°
β = 106.378 (5)°
γ = 107.169 (4)°
*V* = 806.89 (8) Å^3^
*Z* = 2
Mo *K*α radiation
μ = 0.20 mm^−1^
*T* = 296 K
0.72 × 0.64 × 0.58 mm

#### Data collection

Stoe IPDS-2 diffractometer
Absorption correction: integration (*X-RED32*; Stoe & Cie, 2002 ▶) *T*_min_ = 0.867, *T*_max_ = 0.891
17959 measured reflections
3253 independent reflections
2948 reflections with *I* > 2σ(*I*)
*R*_int_ = 0.034

#### Refinement

*R*[*F* ^2^ > 2σ(*F* ^2^)] = 0.046
*wR*(*F* ^2^) = 0.127
*S* = 1.07
3253 reflections
181 parameters
H-atom parameters constrained
Δρ_max_ = 0.71 e Å^−3^
Δρ_min_ = −0.18 e Å^−3^

### 

Data collection: *X-AREA* (Stoe & Cie, 2002 ▶); cell refinement: *X-AREA*; data reduction: *X-RED32* (Stoe & Cie, 2002 ▶); program(s) used to solve structure: *SIR97* (Altomare *et al.*, 1999 ▶); program(s) used to refine structure: *SHELXL97* (Sheldrick, 2008 ▶); molecular graphics: *ORTEP-3 for Windows* (Farrugia, 1997 ▶); software used to prepare material for publication: *WinGX* (Farrugia, 1999 ▶) and *PLATON* (Spek, 2003 ▶).

## Supplementary Material

Crystal structure: contains datablocks global, I. DOI: 10.1107/S1600536808022459/kp2183sup1.cif

Structure factors: contains datablocks I. DOI: 10.1107/S1600536808022459/kp2183Isup2.hkl

Additional supplementary materials:  crystallographic information; 3D view; checkCIF report

## Figures and Tables

**Table 1:** Hydrogen-bond geometry (Å, °)

*D*—H⋯*A*	*D*—H	H⋯*A*	*D*⋯*A*	*D*—H⋯*A*
C5—H5*A*⋯S1	0.97	2.83	3.217 (2)	105
C14—H14*B*⋯O1^i^	0.97	2.65	3.311 (3)	126

Symmetry code: (i) 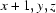.

**Fig. 1.**
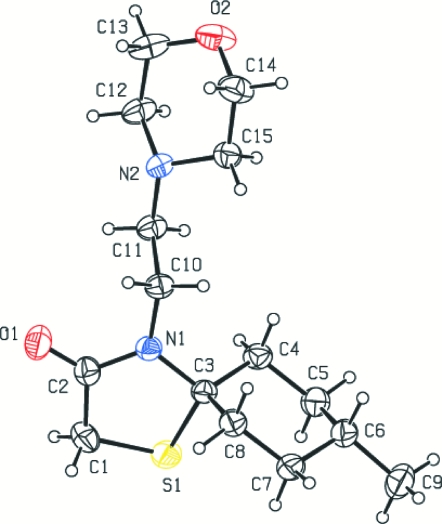
An ORTEP view of the title compound with the atom numbering scheme. Displacement ellipsoids for non-H atoms are drawn at the 30% probability level.

**Fig. 2.**
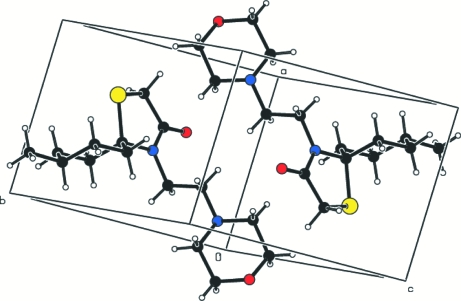
View of the molecular packing in the unit cell.

## supplementary crystallographic information

### Comment

Applications of multi-component reactions (MCRs) in all areas of applied chemistry are very popular because they offer a wealth of products, while requiring only a minimum of effort. The derivatives incorporating the thiazolidine ring system are interesting compounds due to their biological properties. Some 4-thiazolidinones interfere with essential bacterial enzymes (Andres *et al.*, 2000) and they also exhibit antibacterial (Vicini *et al.*, 2006), antimycobacterial (Küçükgüzel *et al.*, 2002), anti-HIV-1 (Barreca *et al.*, 2001), and anticancer activities (Rao *et al.*, 2004). A very recent article deals with similar structures demostrating potent antiproliferative activity for prostate cancer (Gududuru *et al.*, 2004). As a part of an ongoing investigation on bioactive 4-thiazolidinones and related structures, we report here the synthesis and the crystal structure of title compound (I).

In (I) (Fig. 1), all bond lengths and angles are within normal ranges (Allen *et al.*, 1987). The mean C—S bond length [1.816 (2) Å] is larger than the corresponding values in similar molecules [1.778 (2) Å (Akkurt *et al.*, 2008), and 1.737 (5) Å (Akkurt *et al.*, 2007)]. This may be due to the steric interactions among the sulfur and the other atoms around it.

In the title molecule, the five-membered thiazole ring (C1–C3/N1/S1) is in a twisted conformation, with maximum deviations from best least-square plane of -0.131 (1) and 0.143 (1) Å for atoms S1 and C3, respectively. The cyclohexane and morpholine rings (C3–C8) and (C12–C15/N2/O2) have chair conformations with puckering parameters (Cremer & Pople, 1975) Q_T_ = 0.549 (2) Å, θ =178.8 (2) ° and φ = 233 (10) °, and Q_T_ = 0.563 (2) Å, θ = 2.8 (2) ° and φ = 38 (7) °, respectively.

The molecules are stabilized by intramolecular C—H···S interactions, forming a five-membered ring. The packing of the molecules in the unitcell has a significant C14—H14B···O1?C2*^i^* interaction [symmetry code: (i) 1 + *x*, *y*, *z*], where O1 acts as a double acceptor to two symmetrically related H14B [H14B···O1 = 2.65 Å] (Table 1, Fig. 2).

### Experimental

A mixture of morpholinoethylamin (5 mmol), 4-methyl cyclohexanone (5 mmol) and thioglycolicacid (20 mmol) in dry benzene (20 ml) was refluxed for 6 h using a Dean-Stark water separator. Excess solvent was evaporated *in vacuo*. The residue was taken up in chloroform. The chloroform layer was triturated with saturated NaHCO_3_ solution (2x) before drying over sodium sulfate and concentrated under reduced pressure to dryness. The crude product was purified by column chromatography on silica gel using hexane: acetone (80:20) as eluent to yield colourless prisms. IR (ν, cm^-1^): 1672 (C=O). ^1^H-NMR (δ, DMSO-d_6_, 400 MHz): 0.85 (3*H*, d, *J=*6.0 Hz, 8-CH_3_),1.09–1.25 (3*H*, m, cycl. CH), 1.60–1.70 (4*H*, m, cycl. CH), 1.90–2.05 (2*H*, m,cycl.CH), 2.30–2.45 (6*H*, m, morph. N—CH_2_), 3.27 (2*H*, t, *J=*7.6 Hz, N—CH_2_), 3.45 (2*H*, s, SCH_2_), 3.53 (4*H*, t, *J*=4.4 Hz, OCH_2_). LC—MS (m/*z*): 299 (*M*+1). Analysis calculated for C_15_H_26_N_2_O_2_S: C 60.37, H 8.78, N 9.39%. Found: C 59.91, H 8.24, N 9.32%.

### Refinement

All H-atoms were placed in calculated positions [C—H = 0.96–0.97 Å] and were included in the refinement in the riding model approximation, with *U*_iso_(H) set to 1.2 or 1.5 *U*_eq_(C).

The highest residual electron density [0.71 e.A^-3^] was located at 0.86 Å from atom H1A and the deepest residual electron-density [-0.18 e.A^-3^] was located at 0.72 Å from atom S1. Probably due to the poor crystal quality, most of the reflections were weak. Analysis of the solvent void using *PLATON* (Spek, 2003) gave that unit cell contains no residual solvent accessible area.

### Crystal data

**Table:**

C_15_H_26_N_2_O_2_S	*Z* = 2
*M**_r_* = 298.45	*F*_000_ = 324
Triclinic, *P*1	*D*_x_ = 1.228 Mg m^−^^3^
Hall symbol: -P 1	Mo *K*α radiation λ = 0.71073 Å
*a* = 7.8629 (4) Å	Cell parameters from 29201 reflections
*b* = 10.5239 (6) Å	θ = 2.0–28.0º
*c* = 10.8252 (6) Å	µ = 0.20 mm^−^^1^
α = 94.974 (5)º	*T* = 296 K
β = 106.378 (5)º	Block, colourless
γ = 107.169 (4)º	0.72 × 0.64 × 0.58 mm
*V* = 806.89 (8) Å^3^	

### Data collection

**Table:**

Stoe IPDS-2 diffractometer	3253 independent reflections
Monochromator: plane graphite	2948 reflections with *I* > 2σ(*I*)
Detector resolution: 6.67 pixels mm^-1^	*R*_int_ = 0.034
*T* = 296 K	θ_max_ = 26.5º
ω scans	θ_min_ = 2.9º
Absorption correction: integration(X-RED32; Stoe & Cie, 2002)	*h* = −9→9
*T*_min_ = 0.867, *T*_max_ = 0.891	*k* = −13→13
17959 measured reflections	*l* = −13→13

### Refinement

**Table:**

Refinement on *F*^2^	Secondary atom site location: difference Fourier map
Least-squares matrix: full	Hydrogen site location: inferred from neighbouring sites
*R*[*F*^2^ > 2σ(*F*^2^)] = 0.046	H-atom parameters constrained
*wR*(*F*^2^) = 0.127	*w* = 1/[σ^2^(*F*_o_^2^) + (0.07*P*)^2^ + 0.1465*P*] where *P* = (*F*_o_^2^ + 2*F*_c_^2^)/3
*S* = 1.07	(Δ/σ)_max_ < 0.001
3253 reflections	Δρ_max_ = 0.72 e Å^−^^3^
181 parameters	Δρ_min_ = −0.18 e Å^−^^3^
Primary atom site location: structure-invariant direct methods	Extinction correction: none

### Special details

**Table:**

Geometry. Bond distances, angles *etc*. have been calculated using the rounded fractional coordinates. All su's are estimated from the variances of the (full) variance-covariance matrix. The cell e.s.d.'s are taken into account in the estimation of distances, angles and torsion angles
Refinement. Refinement on *F*^2^ for ALL reflections except those flagged by the user for potential systematic errors. Weighted *R*-factors *wR* and all goodnesses of fit *S* are based on *F*^2^, conventional *R*-factors *R* are based on *F*, with *F* set to zero for negative *F*^2^. The observed criterion of *F*^2^ > σ(*F*^2^) is used only for calculating -*R*-factor-obs *etc*. and is not relevant to the choice of reflections for refinement. *R*-factors based on *F*^2^ are statistically about twice as large as those based on *F*, and *R*-factors based on ALL data will be even larger.

### Fractional atomic coordinates and isotropic or equivalent isotropic displacement parameters (Å^2^)

**Table:**

	*x*	*y*	*z*	*U*_iso_*/*U*_eq_	
S1	0.34108 (6)	0.16629 (5)	0.79000 (5)	0.0644 (2)	
O1	0.4404 (2)	0.54993 (14)	0.84353 (15)	0.0737 (5)	
O2	1.1634 (2)	0.77257 (19)	0.54682 (15)	0.0857 (6)	
N1	0.58740 (18)	0.40139 (13)	0.80741 (13)	0.0476 (4)	
N2	0.88645 (18)	0.65537 (12)	0.66493 (12)	0.0465 (4)	
C1	0.2920 (3)	0.3148 (2)	0.8397 (2)	0.0649 (6)	
C2	0.4460 (2)	0.43541 (18)	0.83066 (16)	0.0545 (5)	
C3	0.5878 (2)	0.26243 (15)	0.81122 (14)	0.0444 (4)	
C4	0.6416 (3)	0.20501 (17)	0.69912 (16)	0.0541 (5)	
C5	0.6455 (3)	0.06167 (18)	0.70696 (18)	0.0623 (6)	
C6	0.7743 (3)	0.05266 (18)	0.83773 (19)	0.0592 (6)	
C7	0.7230 (3)	0.11180 (19)	0.94940 (18)	0.0587 (6)	
C8	0.7174 (2)	0.25365 (17)	0.94280 (15)	0.0522 (5)	
C9	0.7691 (4)	−0.0928 (2)	0.8470 (3)	0.0830 (8)	
C10	0.7529 (2)	0.50806 (16)	0.80326 (15)	0.0506 (5)	
C11	0.7251 (2)	0.54594 (17)	0.66860 (16)	0.0530 (5)	
C12	0.8409 (3)	0.7076 (2)	0.54383 (18)	0.0651 (6)	
C13	1.0103 (3)	0.8188 (2)	0.5390 (2)	0.0794 (7)	
C14	1.2124 (3)	0.7255 (3)	0.6657 (2)	0.0766 (7)	
C15	1.0497 (2)	0.61291 (18)	0.67652 (17)	0.0557 (5)	
H1A	0.17090	0.31270	0.78280	0.0780*	
H1B	0.28890	0.31960	0.92890	0.0780*	
H4A	0.76460	0.26320	0.70210	0.0650*	
H4B	0.55170	0.20400	0.61620	0.0650*	
H5A	0.51890	0.00160	0.69320	0.0750*	
H5B	0.68720	0.03110	0.63730	0.0750*	
H6	0.90330	0.10710	0.84630	0.0710*	
H7A	0.60070	0.05350	0.94770	0.0700*	
H7B	0.81400	0.11330	1.03190	0.0700*	
H8A	0.84370	0.31460	0.95710	0.0630*	
H8B	0.67450	0.28300	1.01240	0.0630*	
H9A	0.80140	−0.12910	0.77570	0.1240*	
H9B	0.64500	−0.14730	0.84260	0.1240*	
H9C	0.85780	−0.09330	0.92860	0.1240*	
H10A	0.86080	0.47760	0.82650	0.0610*	
H10B	0.77880	0.58750	0.86740	0.0610*	
H11A	0.70360	0.46730	0.60500	0.0640*	
H11B	0.61440	0.57310	0.64400	0.0640*	
H12A	0.73840	0.74250	0.53870	0.0780*	
H12B	0.80040	0.63490	0.46930	0.0780*	
H13A	0.97770	0.85230	0.45800	0.0950*	
H13B	1.04670	0.89310	0.61120	0.0950*	
H14A	1.25230	0.79980	0.73860	0.0920*	
H14B	1.31720	0.69320	0.67090	0.0920*	
H15A	1.01660	0.53560	0.60800	0.0670*	
H15B	1.08690	0.58510	0.76030	0.0670*	

### Atomic displacement parameters (Å^2^)

**Table:**

	*U*^11^	*U*^22^	*U*^33^	*U*^12^	*U*^13^	*U*^23^
S1	0.0449 (2)	0.0547 (3)	0.0893 (4)	0.0085 (2)	0.0219 (2)	0.0200 (2)
O1	0.0760 (9)	0.0646 (8)	0.0894 (10)	0.0368 (7)	0.0264 (7)	0.0132 (7)
O2	0.0795 (10)	0.1059 (12)	0.0718 (9)	0.0147 (9)	0.0356 (7)	0.0365 (8)
N1	0.0443 (6)	0.0453 (7)	0.0529 (7)	0.0129 (5)	0.0164 (5)	0.0124 (5)
N2	0.0477 (7)	0.0418 (6)	0.0465 (6)	0.0119 (5)	0.0119 (5)	0.0127 (5)
C1	0.0485 (9)	0.0746 (12)	0.0754 (11)	0.0193 (8)	0.0279 (8)	0.0109 (9)
C2	0.0518 (9)	0.0593 (9)	0.0547 (9)	0.0230 (7)	0.0163 (7)	0.0099 (7)
C3	0.0415 (7)	0.0446 (7)	0.0468 (7)	0.0117 (6)	0.0157 (6)	0.0125 (6)
C4	0.0645 (10)	0.0536 (9)	0.0460 (8)	0.0185 (7)	0.0209 (7)	0.0120 (6)
C5	0.0723 (11)	0.0540 (9)	0.0631 (10)	0.0207 (8)	0.0271 (8)	0.0071 (8)
C6	0.0532 (9)	0.0524 (9)	0.0770 (11)	0.0196 (7)	0.0253 (8)	0.0165 (8)
C7	0.0599 (10)	0.0663 (10)	0.0602 (9)	0.0279 (8)	0.0232 (8)	0.0271 (8)
C8	0.0536 (8)	0.0607 (9)	0.0441 (8)	0.0218 (7)	0.0148 (6)	0.0120 (6)
C9	0.0817 (14)	0.0599 (11)	0.1133 (18)	0.0334 (10)	0.0288 (13)	0.0205 (11)
C10	0.0473 (8)	0.0470 (8)	0.0501 (8)	0.0080 (6)	0.0123 (6)	0.0111 (6)
C11	0.0459 (8)	0.0524 (8)	0.0527 (8)	0.0094 (7)	0.0093 (6)	0.0161 (7)
C12	0.0649 (10)	0.0618 (10)	0.0595 (10)	0.0137 (8)	0.0098 (8)	0.0269 (8)
C13	0.0898 (15)	0.0634 (11)	0.0663 (11)	0.0041 (10)	0.0140 (10)	0.0316 (9)
C14	0.0559 (10)	0.0968 (15)	0.0778 (13)	0.0158 (10)	0.0280 (9)	0.0329 (11)
C15	0.0560 (9)	0.0602 (9)	0.0552 (9)	0.0210 (7)	0.0214 (7)	0.0161 (7)

### Geometric parameters (Å, °)

**Table:**

S1—C1	1.791 (2)	C4—H4A	0.9700
S1—C3	1.8410 (17)	C4—H4B	0.9700
O1—C2	1.216 (2)	C5—H5A	0.9700
O2—C13	1.410 (3)	C5—H5B	0.9700
O2—C14	1.412 (3)	C6—H6	0.9800
N1—C2	1.343 (2)	C7—H7A	0.9700
N1—C3	1.468 (2)	C7—H7B	0.9700
N1—C10	1.461 (2)	C8—H8A	0.9700
N2—C11	1.453 (2)	C8—H8B	0.9700
N2—C12	1.458 (2)	C9—H9A	0.9600
N2—C15	1.456 (2)	C9—H9B	0.9600
C1—C2	1.506 (3)	C9—H9C	0.9600
C3—C4	1.526 (3)	C10—H10A	0.9700
C3—C8	1.528 (2)	C10—H10B	0.9700
C4—C5	1.527 (3)	C11—H11A	0.9700
C5—C6	1.520 (3)	C11—H11B	0.9700
C6—C7	1.518 (3)	C12—H12A	0.9700
C6—C9	1.532 (3)	C12—H12B	0.9700
C7—C8	1.514 (3)	C13—H13A	0.9700
C10—C11	1.518 (2)	C13—H13B	0.9700
C12—C13	1.509 (3)	C14—H14A	0.9700
C14—C15	1.506 (3)	C14—H14B	0.9700
C1—H1A	0.9700	C15—H15A	0.9700
C1—H1B	0.9700	C15—H15B	0.9700
			
S1···N1	2.6062 (15)	H5B···H9A	2.5400
S1···H5A	2.8300	H7A···S1	2.8800
S1···H7A	2.8800	H7A···H9B	2.4700
S1···H13B^i^	3.1500	H7A···H7A^viii^	2.2900
S1···H13A^ii^	3.0600	H7B···H9C	2.5200
O1···C11	3.321 (2)	H7B···H9C^ix^	2.5700
O1···C14^iii^	3.311 (3)	H8A···C10	2.8600
O1···C15^iii^	3.380 (2)	H8A···H10A	2.3100
O1···C1^iv^	3.395 (3)	H8B···O1^iv^	2.7600
O1···C2^iv^	3.363 (2)	H9A···N2^x^	2.8100
O2···N2	2.856 (2)	H9A···C12^x^	3.0600
O2···C4^v^	3.419 (3)	H9A···H5B	2.5400
O1···H10B	2.5100	H9B···H5A	2.5500
O1···H15B^iii^	2.8200	H9B···H7A	2.4700
O1···H1B^iv^	2.6900	H9C···H7B	2.5200
O1···H8B^iv^	2.7600	H9C···H7B^ix^	2.5700
O1···H11B	2.8600	H9C···H9C^ix^	2.4900
O1···H14B^iii^	2.6500	H10A···C4	2.8600
N1···S1	2.6062 (15)	H10A···C8	2.8600
N2···O2	2.856 (2)	H10A···C15	2.7200
N2···H9A^vi^	2.8100	H10A···H4A	2.3200
C1···O1^iv^	3.395 (3)	H10A···H8A	2.3100
C2···O1^iv^	3.363 (2)	H10A···H15B	2.1500
C2···C2^iv^	3.565 (2)	H10B···O1	2.5100
C4···C11	3.514 (2)	H10B···H1B^iv^	2.5900
C4···O2^v^	3.419 (3)	H11A···C4	2.9800
C11···O1	3.321 (2)	H11A···H4A	2.5800
C11···C4	3.514 (2)	H11A···H12B	2.4800
C14···O1^vii^	3.311 (3)	H11A···H15A	2.3400
C15···O1^vii^	3.380 (2)	H11B···O1	2.8600
C2···H11B	2.9700	H11B···C2	2.9700
C4···H11A	2.9800	H11B···H12A	2.3200
C4···H10A	2.8600	H12A···H11B	2.3200
C8···H10A	2.8600	H12B···H11A	2.4800
C10···H15B	2.7000	H12B···H15A	2.4700
C10···H8A	2.8600	H13A···S1^ii^	3.0600
C10···H4A	2.7500	H13B···S1^xi^	3.1500
C12···H9A^vi^	3.0600	H13B···C15	3.1000
C15···H10A	2.7200	H13B···H14A	2.3200
C15···H13B	3.1000	H14A···H13B	2.3200
H1B···O1^iv^	2.6900	H14B···O1^vii^	2.6500
H1B···H10B^iv^	2.5900	H15A···H11A	2.3400
H4A···C10	2.7500	H15A···H12B	2.4700
H4A···H10A	2.3200	H15A···H15A^v^	2.3100
H4A···H11A	2.5800	H15B···O1^vii^	2.8200
H5A···S1	2.8300	H15B···C10	2.7000
H5A···H9B	2.5500	H15B···H10A	2.1500
			
C1—S1—C3	92.98 (9)	C9—C6—H6	108.00
C13—O2—C14	109.27 (17)	C6—C7—H7A	109.00
C2—N1—C3	118.93 (14)	C6—C7—H7B	109.00
C2—N1—C10	119.11 (14)	C8—C7—H7A	109.00
C3—N1—C10	120.98 (14)	C8—C7—H7B	109.00
C11—N2—C12	110.57 (14)	H7A—C7—H7B	108.00
C11—N2—C15	112.08 (13)	C3—C8—H8A	109.00
C12—N2—C15	109.11 (15)	C3—C8—H8B	109.00
S1—C1—C2	107.46 (16)	C7—C8—H8A	109.00
O1—C2—N1	124.77 (17)	C7—C8—H8B	109.00
O1—C2—C1	122.89 (18)	H8A—C8—H8B	108.00
N1—C2—C1	112.34 (16)	C6—C9—H9A	110.00
S1—C3—N1	103.37 (11)	C6—C9—H9B	109.00
S1—C3—C4	108.79 (12)	C6—C9—H9C	109.00
S1—C3—C8	110.62 (11)	H9A—C9—H9B	109.00
N1—C3—C4	112.65 (13)	H9A—C9—H9C	109.00
N1—C3—C8	111.03 (12)	H9B—C9—H9C	109.00
C4—C3—C8	110.18 (14)	N1—C10—H10A	109.00
C3—C4—C5	111.69 (15)	N1—C10—H10B	109.00
C4—C5—C6	112.69 (15)	C11—C10—H10A	109.00
C5—C6—C7	110.18 (19)	C11—C10—H10B	109.00
C5—C6—C9	112.17 (18)	H10A—C10—H10B	108.00
C7—C6—C9	110.37 (18)	N2—C11—H11A	109.00
C6—C7—C8	113.08 (15)	N2—C11—H11B	109.00
C3—C8—C7	112.40 (14)	C10—C11—H11A	109.00
N1—C10—C11	111.79 (13)	C10—C11—H11B	109.00
N2—C11—C10	112.12 (13)	H11A—C11—H11B	108.00
N2—C12—C13	110.35 (17)	N2—C12—H12A	110.00
O2—C13—C12	111.52 (18)	N2—C12—H12B	110.00
O2—C14—C15	111.56 (18)	C13—C12—H12A	110.00
N2—C15—C14	111.32 (17)	C13—C12—H12B	110.00
S1—C1—H1A	110.00	H12A—C12—H12B	108.00
S1—C1—H1B	110.00	O2—C13—H13A	109.00
C2—C1—H1A	110.00	O2—C13—H13B	109.00
C2—C1—H1B	110.00	C12—C13—H13A	109.00
H1A—C1—H1B	109.00	C12—C13—H13B	109.00
C3—C4—H4A	109.00	H13A—C13—H13B	108.00
C3—C4—H4B	109.00	O2—C14—H14A	109.00
C5—C4—H4A	109.00	O2—C14—H14B	109.00
C5—C4—H4B	109.00	C15—C14—H14A	109.00
H4A—C4—H4B	108.00	C15—C14—H14B	109.00
C4—C5—H5A	109.00	H14A—C14—H14B	108.00
C4—C5—H5B	109.00	N2—C15—H15A	109.00
C6—C5—H5A	109.00	N2—C15—H15B	109.00
C6—C5—H5B	109.00	C14—C15—H15A	109.00
H5A—C5—H5B	108.00	C14—C15—H15B	109.00
C5—C6—H6	108.00	H15A—C15—H15B	108.00
C7—C6—H6	108.00		
			
C3—S1—C1—C2	17.14 (14)	C12—N2—C15—C14	54.33 (18)
C1—S1—C3—C4	−140.08 (12)	C12—N2—C11—C10	−168.37 (15)
C1—S1—C3—C8	98.77 (13)	C15—N2—C12—C13	−54.9 (2)
C1—S1—C3—N1	−20.15 (11)	S1—C1—C2—O1	171.24 (15)
C13—O2—C14—C15	58.7 (2)	S1—C1—C2—N1	−8.90 (19)
C14—O2—C13—C12	−59.8 (2)	N1—C3—C8—C7	−179.28 (15)
C3—N1—C2—O1	172.02 (16)	C8—C3—C4—C5	54.1 (2)
C10—N1—C2—O1	3.3 (2)	S1—C3—C8—C7	66.56 (17)
C10—N1—C3—C8	69.70 (18)	N1—C3—C4—C5	178.67 (16)
C2—N1—C3—C4	137.07 (16)	C4—C3—C8—C7	−53.77 (19)
C2—N1—C3—S1	19.82 (16)	S1—C3—C4—C5	−67.33 (18)
C10—N1—C3—S1	−171.66 (11)	C3—C4—C5—C6	−55.4 (2)
C10—N1—C2—C1	−176.58 (14)	C4—C5—C6—C7	53.7 (2)
C10—N1—C3—C4	−54.41 (19)	C4—C5—C6—C9	177.0 (2)
C2—N1—C3—C8	−98.82 (17)	C5—C6—C7—C8	−53.1 (2)
C3—N1—C2—C1	−7.8 (2)	C9—C6—C7—C8	−177.6 (2)
C3—N1—C10—C11	106.22 (16)	C6—C7—C8—C3	54.4 (2)
C2—N1—C10—C11	−85.28 (18)	N1—C10—C11—N2	177.91 (13)
C11—N2—C15—C14	177.14 (14)	N2—C12—C13—O2	59.0 (2)
C11—N2—C12—C13	−178.59 (16)	O2—C14—C15—N2	−57.2 (2)
C15—N2—C11—C10	69.65 (17)		

Symmetry codes: (i) *x*−1, *y*−1, *z*; (ii) −*x*+1, −*y*+1, −*z*+1; (iii) *x*−1, *y*, *z*; (iv) −*x*+1, −*y*+1, −*z*+2; (v) −*x*+2, −*y*+1, −*z*+1; (vi) *x*, *y*+1, *z*; (vii) *x*+1, *y*, *z*; (viii) −*x*+1, −*y*, −*z*+2; (ix) −*x*+2, −*y*, −*z*+2; (x) *x*, *y*−1, *z*; (xi) *x*+1, *y*+1, *z*.

### Hydrogen-bond geometry (Å, °)

**Table:**

*D*—H···*A*	*D*—H	H···*A*	*D*···*A*	*D*—H···*A*
C5—H5A···S1	0.97	2.83	3.217 (2)	105
C14—H14B···O1^vii^	0.97	2.65	3.311 (3)	126

Symmetry codes: (vii) *x*+1, *y*, *z*.
